# Supplementary figures and images for: Carbapenem-resistant Enterobacter hormaechei uses mucus metabolism to facilitate gastrointestinal colonization
Source: mBio. 2025 Jan 29;16(3):e02884-24. doi: 10.1128/mbio.02884-24 (PMC11898723; doi:10.1128/mbio.02884-24)

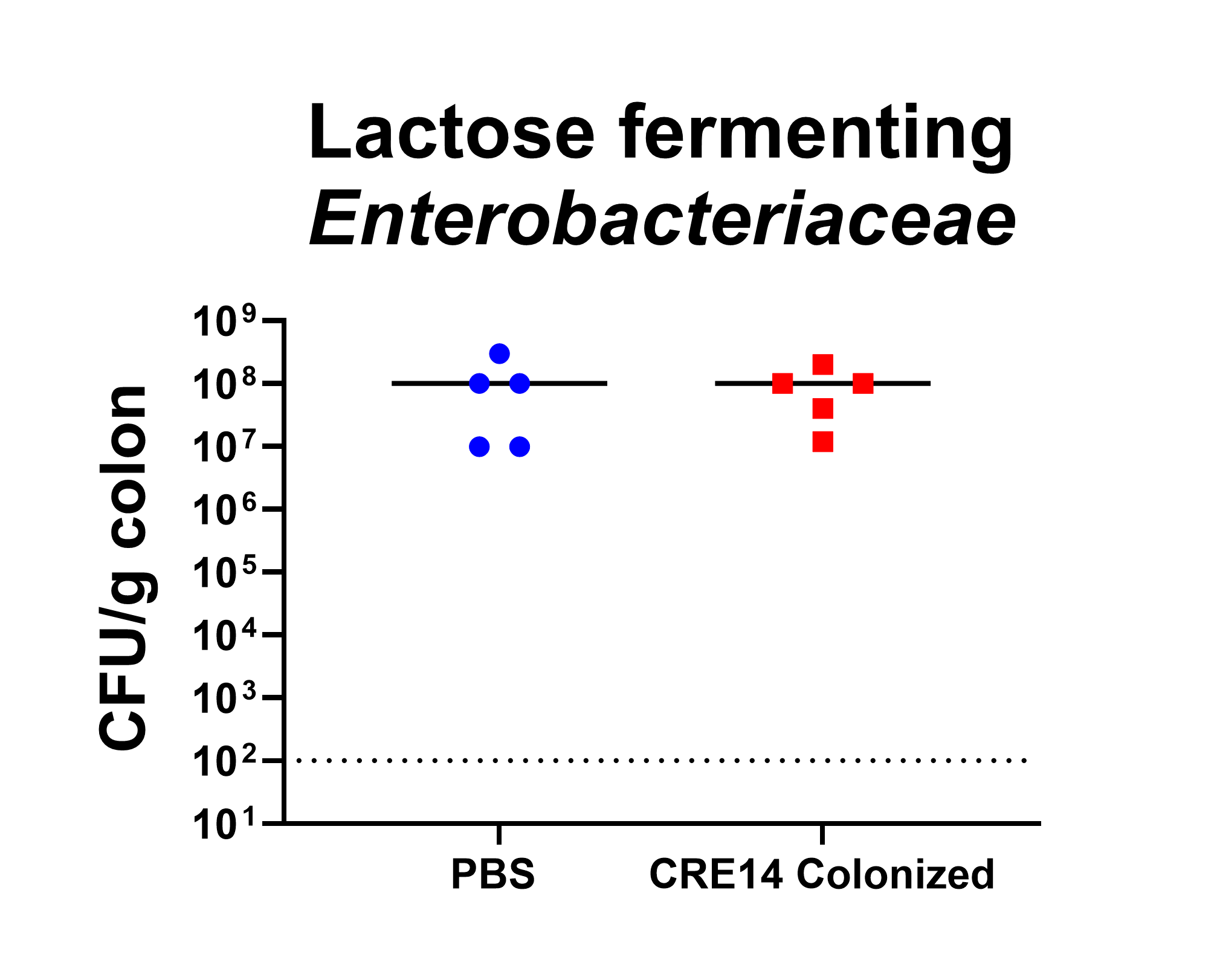

Supplement: Fig. S1 — Quantification of endogenous lactose-fermenting E. coli in PBS and CRE14 colonized infant mouse colons. [file mbio.02884-24-s0001.tif]

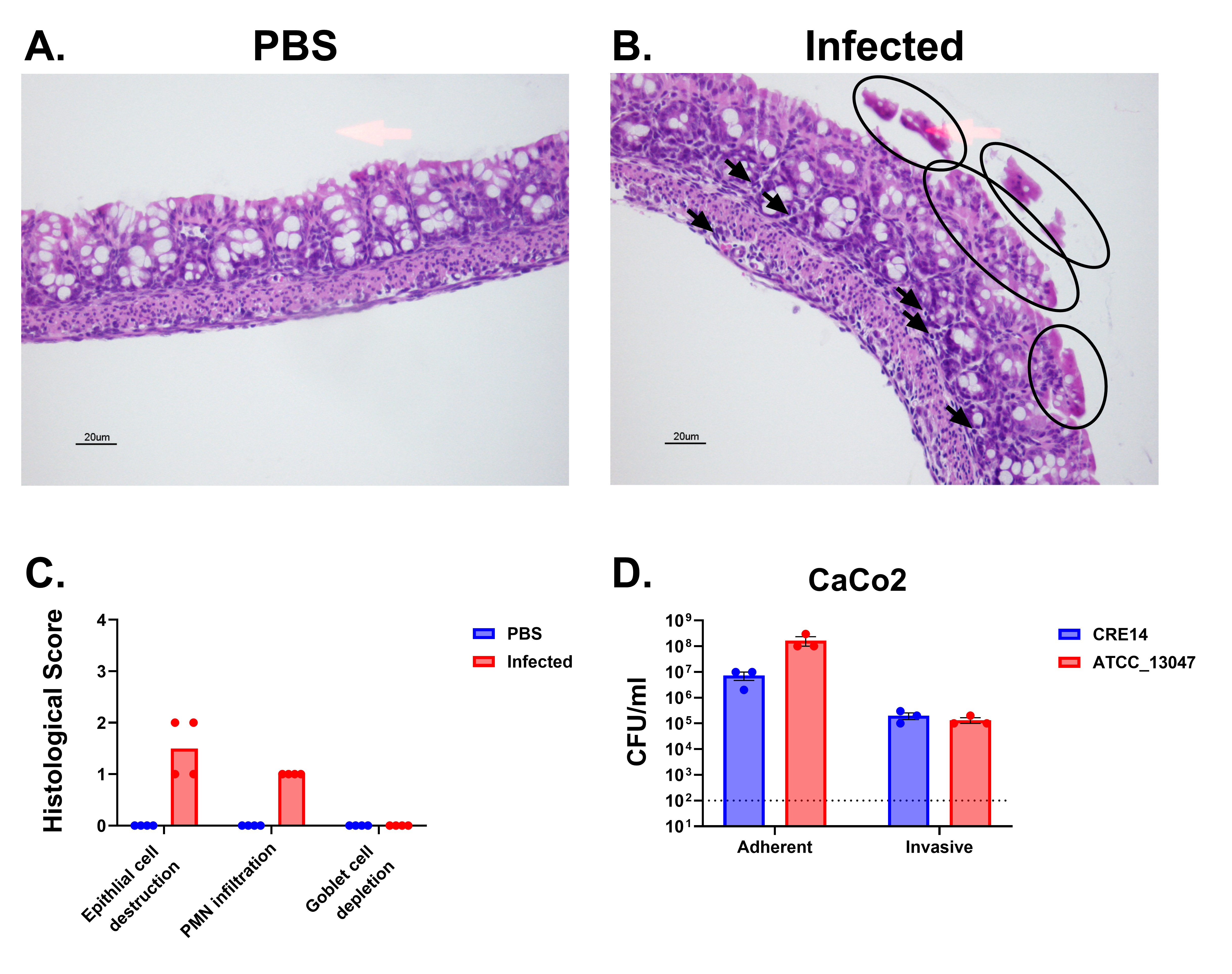

Supplement: Fig. S2 — H&E staining of colon sections from PBS or CRE14 colonized CD-1 infant mice. [file mbio.02884-24-s0002.tif]

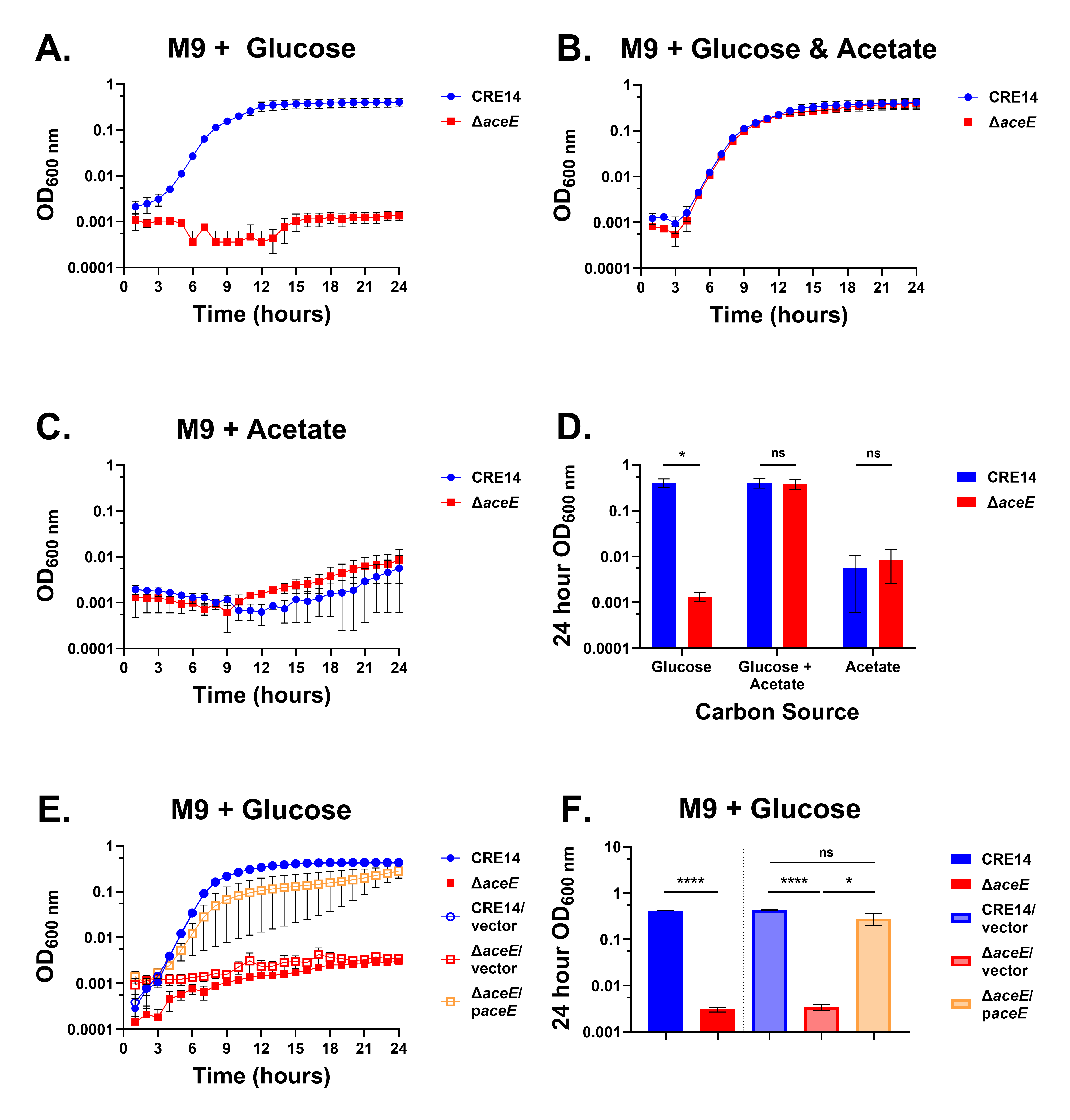

Supplement: Fig. S3 — Growth of CRE14 and ΔaceE in M9 minimal medium containing glucose, acetate, or glucose and acetate. [file mbio.02884-24-s0003.tif]

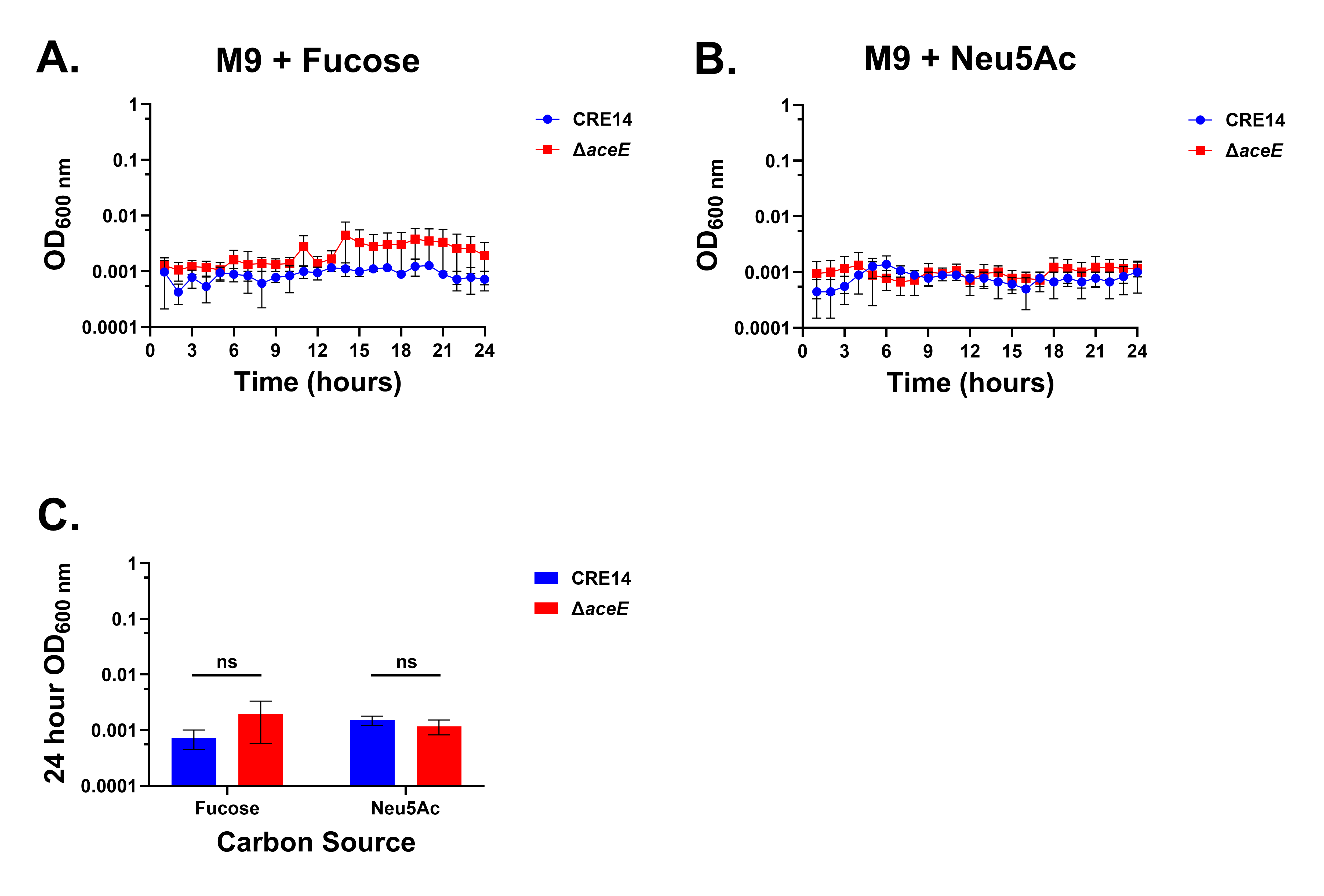

Supplement: Fig. S4 — Growth of CRE14 and ΔaceE in M9 minimal medium containing fucose or sialic acid. [file mbio.02884-24-s0004.tif]

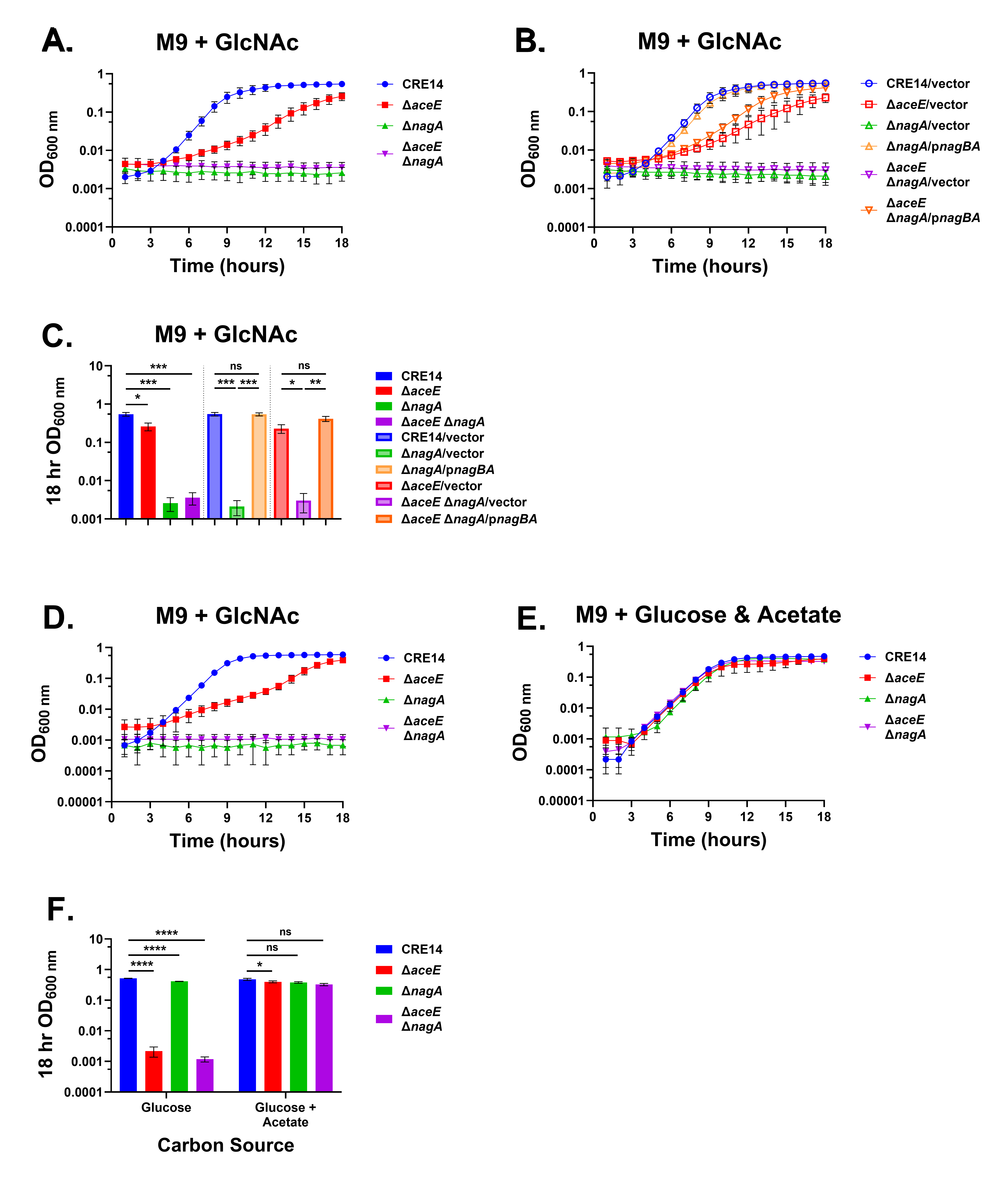

Supplement: Fig. S5 — Complementation of ΔnagA mutant or ΔaceE mutant. [file mbio.02884-24-s0005.tif]
