# Supplementary material for: Carbapenem-resistant Enterobacter hormaechei uses mucus metabolism to facilitate gastrointestinal colonization
Source: mBio. 2025 Jan 29;16(3):e02884-24. doi: 10.1128/mbio.02884-24 (PMC11898723; doi:10.1128/mbio.02884-24)
Supplement: Legends — Supplemental figure legends. [file mbio.02884-24-s0006.docx]

**Supplemental Figure Legends**

**Supplemental Figure 1.**  Quantification of endogenous lactose fermenting *E. coli* in PBS and CRE14 colonized infant mouse colons. Each point represents the bacterial burden recovered from the colon of an individual mouse. Horizontal lines represent the median CFU/g for each group, and dashed lines represent the limit of detection.

**Supplemental Figure 2.**  H&E staining of colon sections from PBS (**A**) or CRE14 colonized (**B**) CD-1 infant mice. Circles indicate areas of epithelial cell destruction, and arrows indicate evidence of PMN infiltration. **C**) Histological scoring of H&E stained sections. **D**) Quantification of adherent and invasive CRE14 and ATCC_13047.

**Supplemental Figure 3. A-D**) Growth of CRE14 and Δ*aceE* in M9 minimal medium containing 10 mM glucose (**A**), acetate (**B**), or glucose and acetate (**C**). **D**) Final OD_600nm_ of strains after 24 hours of growth in glucose, acetate, or glucose plus acetate. **E**) Complementation of Δ*aceE* in M9 minimal medium containing 10 mM glucose. **F**) Final OD_600nm_ of strains from (**E**). Data represent the mean of three biological experiments and error bars represent the SEM. Statistical analysis was determined by t-test for the 24-hour timepoint (* p<0.05; ** p<0.01; *** p<0.001; **** p<0.0001).

**Supplemental Figure 4.** Growth of CRE14 and Δ*aceE* in M9 minimal medium containing 10 mM fructose (**A**) or sialic acid (**B**). **C**) Final OD_600nm_ of strains after 24 hours of growth in indicated carbon sources. Data represent the mean of three biological replicates and error bars represent the SEM. Statistical significance was determined by unpaired t-test (* p<0.05; ** p<0.01; *** p<0.001; **** p<0.0001).

**Supplemental Figure 5. A-C**) Complementation of Δ*nagA* mutant. Growth of CRE14, Δ*aceE*, Δ*nagA*, and Δ*aceE* Δ*nagA* (**A**) or empty vector and complemented strains (**B**) in M9 minimal medium containing GlcNAc for 24 hours. **C**) Final OD_600nm_ of strains after 24 hours of growth in M9 minimal medium containing GlcNAc. **D-F**) Chemical complementation of the Δ*aceE* mutant. Growth of CRE14, Δ*aceE*, Δ*nagA*, and Δ*aceE* Δ*nagA* in M9 minimal medium containing 10 mM glucose (**D**) or glucose and acetate (**E**). **F**) Final OD_600nm of_ strains after 24 hours of growth in indicated carbon sources. Data represent the mean OD_600nm of_ three biological replicates and error bars represent the SEM. Statistical significance was determined by unpaired t-test for the 24-hour timepoint (* p<0.05; ** p<0.01; *** p<0.001; **** p<0.0001).
