# Supplementary material for: Carbapenem-resistant Enterobacter hormaechei uses mucus metabolism to facilitate gastrointestinal colonization
Source: mBio. 2025 Jan 29;16(3):e02884-24. doi: 10.1128/mbio.02884-24 (PMC11898723; doi:10.1128/mbio.02884-24)
Supplement: Supplemental Tables — Tables S1 and S2. [file mbio.02884-24-s0007.pdf]

Supplemental Tables:

Supplemental Table 1: Primers

Name	Sequence (5' to 3')	Description
KO_aceE_F	tccagacaaatcaacgtattagatagataaggaataccccatgtcagaagacgatcgaattggggatctt	Amplify *acc(3)IV* resistance cassette with homology to up- and down-stream of *aceE* (PQF87_02045)
KO_aceE_R	gtccggtacattgatttcgatagccattattcttttacctcttacgccaggcatgatcgaattagcttcaaaag	
aceE_screen_F	tccagacaaatcaacgtattagatagataaggaataccccatgtcagaagacgatcgaattggggatctt	Screen for successful insertion-deletion at the *aceE* locus
aceE_screen_R	gtccggtacattgatttcgatagccattattcttttacctcttacgccaggcatgatcgaattagcttcaaaag	
KO_nagA_F	attaccgatttgagcttgtccgcctggtgtcatacttttacttactcagtgacgatcgaattggggatctt	Amplify *acc(3)IV* resistance cassette with homology to up- and down-stream of *nagA* (PQF87_04830)
KO_nagA_R	cttttatccggggcggtcgcttttttaaccgggggtcgatatgtacgctgcatgatcgaattagcttcaaaag	
nagA_screen_F	tccgtgacgatggaaatc	Screen for successful insertion-deletion at the *nagA* locus
nagA_screen_R	ctggatgccgaagaagtg	
pBBR1_atbR_F	cgtctcattttcgccaaaag	Inverse PCR of pBBR1MCS from the *cat* locus
pBBR1_atbR_R	atgaccatgattacgccaag	
HygR_F	cttggcgtaatcatggtcatctattcctttgccctcgg	Amplify the hygromycin resistance cassette with homology to pBBR1MCS from pSIM18
HygR_R	cttttggcgaaaatgagacgcattcaaatatgtatccgctcatg	
pBBR1_atbR_screen_F	actatagggcgaattggagc	Screen for successful insertion of HygR in the pBBR1 backbone
pBBR1_atbR_screen_R	ctgacgtctaagaaaccattattatcatg	
pBBR1Hyg_MCS_F	caggaattcgatatcaagcttatcg	Inverse PCR of pBBR1Hyg at the multi-cloning site
pBBR1Hyg_MCS_R	ctagttctagagcggccg	
pBBR1Hyg_MCS_screen_F	gtcacgacgttgtaaaacgac	Screen for successful insertion at the pBBR1Hyg multi-cloning site
pBBR1Hyg_MCS_screen_R	cgcaattaaccctcactaaagg	
aceE_comp_F	atgagctcactagtggatccctcgtgtgtggttgttatg	Amplify wild-type *pdhR-aceE* alleles with homology to pBBR1Hyg
aceE_comp_R	tcgcgaggtaccgggcccaagtccggtacattgatttcgatag	
nagA_comp_F	agcttgatatcgaattcctgcttctttgatcaggccgc	Amplify wild-type *nagBA* alleles with homology to pBBR1Hyg
nagA_comp_R	ggcggccgctctagaactagcctattccccctacgagac	

Supplemental Table 2: Plasmids

Name	AtbR	Description	Citation
pUC18-miniTn7T-apra	AmpR,<br>ApraR	MiniTn7 vector. Template for *acc(3)IV* apramycin resistance cassette.	(42)
pSIM18	HygR,<br>30°C	Lambda Red recombineering plasmid. pSC101 Ori. Lambda *exo, beta,* and *gam* under expression of CI87 temperature sensitive repressor.	(43)
pCP20	CmR<br>30°C	Flp recombinase plasmid.	(44)
pBBR1MCS	CmR	Broad host range complementation vector that is stable without selection.	(45)
pBBR1Hyg	HygR	Hygromycin- resistant derivative of pBBR1MCS complementation vector.	This study
pBBR1Hyg-*pdhR*-*aceE*	HygR	Vector for complementation of the Δ*aceE* mutation. Contains wild-type *pdhR* and *aceE* alleles with their native promoters.	This study
pBBR1Hyg-*nagBA*	HygR	Vector for complementation of the Δ*nagA* mutation. Contains wild-type *nagBA* alleles and their native promoter.	This study
